# SPECT/CT-plethysmography – non-invasive quantitation of bone and soft tissue blood flow

**DOI:** 10.1186/1749-799X-3-36

**Published:** 2008-08-18

**Authors:** Lior Dayan, Zohar Keidar, Ora Israel, Victor Milloul, Johnathan Sachs, Giris Jacob

**Affiliations:** 1 Ortopedic B and Recanati Autonomic Dysfunction Center, Rambam Health Care Campus, Haifa, Israel; 2 Nuclear Medicine department, Rambam Health Care Campus & The Technion Faculty of Medicine, Haifa, Israel; 3 Rambam Health Care Campus & The Technion Faculty of Medicine – IIT, Haifa, Israel; 4 Director, J. Recanati Autonomic Dysfunction Center, Medicine A, Rambam Health Care Campus & The Technion Faculty of Medicine – IIT, Haifa, Israel

## Abstract

Preserved blood flow to bone and soft tissue is essential for their normal function. To date only numerous methods are suitable for direct bone blood flow (BBF) measurement. Here, we introduce a novel quantitative method for bone and soft tissue blood flow (BBF and SBF, respectively) measurement. It involves a combination of SPECT/CT imaging for blood pool localization in a specific region of interest ("soft" and "hard" tissues composing a limb) with veno-occlusive plethysmography. Using it, we measured BBF and SBF in the four limbs of 10 healthy subjects. At steady state blood flow measurements in the four limbs were similar, ranging between 5.5 – 6.5 and 1.87–2.48 ml per 100 ml of tissue per minute for BBF and SBF, respectively. Our results are comparable to those in the literature. We concluded that SPECT/CT-plethysmography appears to be a readily available and easy to use method to measure BBF and SBF, and can be added to the armamentarium of methods for BBF measurements.

## Introduction

As with all organs, bone blood flow (BBF) is vital to ongoing skeletal function and growth. BBF preserved at a sufficient degree is a crucial component of normal bone turnover and contributes significantly to the basic metabolic processes preserving bone integrity, as well as to repair mechanisms in pathological conditions such as fractures, infections and osteoporosis [1].

Since blood flow to every organ is a dynamic process regulated by internal and external systems, its investigation requires methods that are accurate and reproducible. One method is the positron emission tomography (PET), which is a powerful and widely accepted tool in skeletal muscle perfusion study in humans [2,3]. It has also been utilized for BBF measurement in human [4,5,3], yet this method requires the availability of radioactive substances with extremely short half-life time (e.g. ^15^O nuclide), which are not readily available in many medical centers, thus rendering it unavailable for routine BBF measurements. Other acceptable methods for BBF assessment are either invasive or involve noninvasive imaging techniques with visual non-quantitative assessment of the transit of various radiotracers through certain anatomical region [6-9]. Laser Doppler technique, one of the most currently acknowledged and accepted techniques for use in BBF measurement in humans, is invasive and provides only a qualitative assessment of BBF. Additional methods of BBF measurement, although accurate and validated in many researches, were developed mainly for animal research (e.g., labeled microspheres, thermocouples and dilution methods) and are not applicable for use in humans [10,7,17].

Given the paucity of a suitable quantitative methods for BBF measurement in humans, we were encouraged to develop one that would be relatively safe and available. Using dual modality SPECT/CT imaging devices, it is possible to accurately localize as well as quantify blood pool in regions of interest, i.e within limb compartments. The current study presents a novel method that is based on the combination of two well known clinical tools, strain gauge plethysmography and dual modality SPECT/CT functional and anatomic imaging. The first component of this method enables blood flow measurement in an entire limb. The second enables the highly accurate localization of radiotracer activity to a specific region of interest [18], in our case blood pool in limb different limb's compartments.

## Methods

### Subjects

A group of 10 (4 females and 6 males) healthy subjects aged between 20 and 45, without any history of previous limb fractures, soft tissue damage or major trauma, diseases affecting the vascular system and with no history of any routine medication intake were recruited for the research. No smoking or alcoholic and monoamine containing beverages were allowed 24 hours prior the study. All subjects signed informed and written consent forms approved by the local institutional ethics committee.

### Experimental design

Plethysmography studies were performed in a quiet, darkened room with ambient temperature of ~24°C and following an overnight fast. Thereafter, SPECT/CT studies were acquired. Both studies were performed in a supine position after a 10 minutes supine rest.

## Plethysmography studies

Limb blood flow (F_L_) was measured using the well acknowledged venous occlusion plethysmography technique[19]. Briefly, a sphygmomanometer cuff was applied at a predetermined point in the limb under investigation (i.e. 10 cm distal to the tibial tubercle in the leg and 10 cm distal to the olecranon tip in the forearm) and was inflated to 45 mm Hg for 7 seconds to prevent venous egress. During this period, forearm volume changes per time unit (correlates with blood flow changes) were measured by a strain gauge plethysmography (ECR5, Hokanson, Inc, Bellevue WA, USA). A 7-second deflation period was allowed before the subsequent measurement. The flow to the hand and foot was excluded by inflating a cuff above the systolic BP in the wrist or ankle, respectively. Baseline blood flow was the average of at least 4 stable repeated flow measurements. In order to test the reproducibility of the introduced method, all four limbs were measured in the same session. Each limb was taken as control for its contralateral. It is important to note that the plethysmography method measures the whole limb blood. While apparently it is the soft tissue volume that is changed in response to venous occlusion, it is the venous vasculature congestion within the soft tissue rather than soft tissue congestion per-se that is responsible for the volume changes that allow us to measure the blood flow.

### Quantitative SPECT/CT scintigraphy

Immediately following the plethysmography study, a SPECT/CT study of the upper and lower limbs was performed on all patients 10 minutes following intravenous administration of 740 MBq Tc99m in-vitro labeled red blood cells[20]. SPECT/CT was performed using a nuclear medicine dual head variable angle gamma camera system equipped with a low power x-ray imaging system (Infinia & Hawkeye, GE Healthcare Technologies, USA).

The x-ray imaging system is composed of an x-ray tube and a set of detectors located opposite the x-ray tube. They are mounted on the same gantry and rotate around the patient with the gamma detectors. SPECT and CT scan acquired sequentially with the patient remaining completely still between the scans. Resolution of the x-ray image is 1 mm, but localization images used for clinical reading are produced with a 1.69 mm pixel size. The x-ray images are acquired and reconstructed using the integrated workstation. The data is then transferred to the nuclear medicine database of the processing workstation (Xeleris, GE Healthcare Technologies, USA). SPECT images were acquired using a dual energy window session providing emission and scatter emission projection. The emission acquisition protocol was performed using a matrix size of 128 × 128, parallel head configuration, 180 degrees rotation per head, with an angle step of 3 degrees. Time per frame was 25 seconds. Reconstruction of SPECT data was performed on the processing workstation using scatter correction and attenuation correction (based on attenuation maps derived from the CT image). CT was also used as anatomical map for the functional NM data. The radiation dose to the patient (i.e. the combination of the radiation dose from the SPECT radiopharmaceutical and the radiation dose from the CT portion of the study) was estimated to 6 mS_V_.

#### Calculations and statistical analysis

##### Blood flow calculations

**SPECT/CT data (volume and scintigraphic readings)**

**Figure 1:**
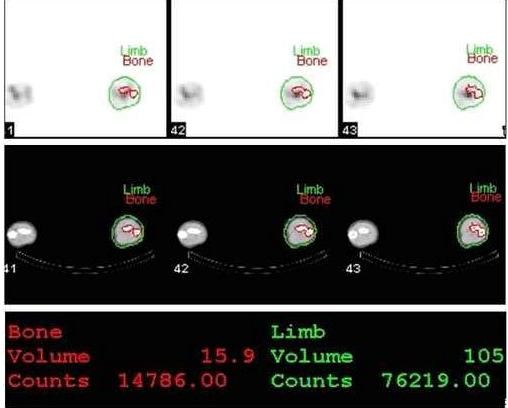
SPECT/CT reconstruction with X-ray image showing volume (in ml) and counts in the bone (red) and total limb (green).

1. Volumes and blood pool activity of the bone (including bone marrow) and soft tissue were determined using segmentation based on thresholds within a virtual cylinder consistent of 3–4 slices (slice thickness 7 mm) on the CT image. The height of the virtual cylinder on which measurements were performed was of approximately 1.4 – 2.1 cm. Precise calculation of the entire cylinder volume (V_L_) is provided by the CT component of the dual modality imaging procedure (see additional file 2).

2. Bone volumes, including the bone marrow (V_B_), were derived from the CT scan using an in-house software that performs segmentation of the bone and soft tissue for each CT slice, subsequently creating corresponding regions of interest. The regions of interest are copied to the registered reformatted SPECT slices in order to correspond to CT voxel size (Figure 1).

3. Data regarding total limb and bone blood pool confined to the virtual cylinder (R_L _and R_B _respectively) was derived from scintigraphic data using the corresponding counts confined to V_L _and V_B _(raw data shown in additional file 3).

4. The limb (and thus the)virtual cylinder is composed of soft tissue (mainly muscles and skin) and "hard" tissue (bone and bone marrow). The soft tissue volume (V_S_) and blood pool (R_S_) are calculated as follows: V_S _= V_L_-V_B _and the corresponding reading R_S _= R_L_-R_B_.

Bone and soft tissue blood flow measurements (F_B _and F_S_, respectively) are based on the following considerations:

1. Assume that a part of the leg or arm under examination is in a form of a cylinder.

The cylinder is composed of two compartments: the bony compartment and the soft tissue compartment.

2. We define the blood volumes (units are ml blood) within each compartment:

υ_B _– blood volume within bone compartment (including bone marrow).

υ_s _– blood volume within soft tissue compartment.

**υ**_**L **_– blood volume within the volume that is confined within the 100 ml cylinder.

3. Based upon plethysmographic measurements, blood flow to the limb (in the selected area) is:

F_L= _υ_L_/min·100 ml tissue (ml blood/min·100 ml tissue)

If we determine the portion of limb under examination (i.e. the cylinder) volume is 100 ml, then: υ_L _ml blood pass through it in 1 minute.

4. The main assumption is that in a resting state, the vasoregulatory systems are balanced, thus the blood flow in each compartment is constant, and the momentary blood flow can be calculated from the plethysmography. Say that a momentary blood flow through the 100 ml cylinder occurs within a time period dt(t→0), then:

υ_L_(dt) = υ_L_·dt/min (note that dt and min are both time units, thus υ_L_dt units are volume units – i.e. ml blood. It means that a momentary volume of υ_L_·dt/min pass during a dt period of time)

5. Since only RBC are marked, the scintigraphic readings are proportional to the blood pool within each compartment.

Say that:

R_B _– scintigraphic reading within the bony compartment in the virtual cylinder.

R_S _– scintigraphic reading within the soft tissue compartment.

R_L _– scintigraphic reading within the entire limb compartment.

6. During the infinitesimal time period **dt**, the blood volume within the bony compartment are proportional to the scintigraphic readings.

υ_B(dt) _= (R_B_/R_L_)·υ_L_·dt/min   (units are of volume-i.e ml blood)

7. We can measure bone and soft tissue compartments volumes precisely from the CT scans: we take the 3–4 slices within the virtual cylindered shape limb portion under examination. We know the slice thickness (slice thickness 7 mm – the distance between the CT slices). The height of a virtual cylinder that its cross sectional area is equal to the slices' is 1.4 – 2.1 cm.

8. The mean measured cylinder volume between the CT slices (V_L_) is 149 ml and 260 ml for the upper and lower limbs, respectively (see additional file 2). Since these volumes are quite small, we may say that within a 100 ml piece of this virtual cylinder the ratios between the bone and soft tissue volumes is preserved.

9. Say that V_B_/V_L _is the relative bone volume of the virtual cylinder between the slices. Thus, in order to calculate the **momentary **blood volume within a 100 ml **bony**compartment (υ_B_(dt)_100_), we need to multiply υ_B_(dt) by the ratio V_L_/V_B_:

In this way:

$$\upsilon_{\text{B}}{\left(\text{dt}\right)}_{100}=\frac{\upsilon_{\text{B}}(\text{dt}){\text{V}}_{\text{L}}}{{\text{V}}_{\text{B}}}=\frac{({\text{R}}_{\text{B}}/{\text{R}}_{\text{L}})\cdot \upsilon_{\text{L}}\text{dt}/\min\cdot {\text{V}}_{\text{L}}}{{\text{V}}_{\text{B}}}=\frac{({\text{R}}_{\text{B}}/{\text{R}}_{\text{L}})\cdot \upsilon_{\text{L}}\cdot \text{dt}/\min\cdot {\text{V}}_{\text{L}}}{{\text{V}}_{\text{B}}}$$

10. If we assume, again, that in resting position the vasoregulatory systems are in balance, and the ratios V_L_/V_B_; υ_L_/V_L_; and R_B_/R_L _remain constant, then we can correct to a minute flow by multiplying υ_B_(dt)_100 _min/dt, which gives:

$${\text{F}}_{\text{B}}=\frac{\upsilon \text{B}(\min\cdot 100)}{{\text{V}}_{\text{B}}}=\frac{\upsilon \text{L}\cdot ({\text{R}}_{\text{B}}/{\text{R}}_{\text{L}})\cdot {\text{V}}_{\text{L}}}{{\text{V}}_{\text{B}}}$$

11. In a same way, the soft tissue blood flow per 100 ml soft tissue per minute is:

$${\text{F}}_{\text{s}}=\frac{\upsilon_{\text{S}}(\min\cdot 100)}{V_{S}}=\frac{\upsilon_{\text{L}}\cdot ({\text{R}}_{\text{S}}/{\text{R}}_{\text{L}})\cdot {\text{V}}_{\text{L}}}{V_{S}}$$

### Statistical Analysis

Data are presented as mean ± SEM. Wilcoxon-matched-paired test, which is suitable for comparison between small groups, was used to compare between upper and lower limbs and their contralaterals. The level selected for statistical significance was set at P value < 0.05. Data were analyzed with Excel (Microsoft 2000, USA) and GraphPad Prism (version 3.0, GraphPad Softwarte, Inc., San Diego, CA).

## Results

Six men and four women were evaluated. Subject's mean age, weight, height, body mass index (BMI, weight/height in m^2^), systolic and diastolic blood pressure and heart rates are presented in additional file 1. Raw volume measurements and scintigraphic readings depicted from SPECT/CT are shown in additional file 2. Briefly, the limbs' parts volumes that were examined (referred as a "virtual cylinder" in the methods section) were 149 ± 15, 149 ± 16 ml for right and left upper limbs, and 265 ± 22 and 256 ± 20 ml for the right and left lower limbs, respectively.

At steady state blood flow measurements in the four limbs ranged between 5.5 – 6.5 and 1.87–2.48 ml per 100 ml of tissue per minute for BBF and SBF, respectively.

**Figure 2:**
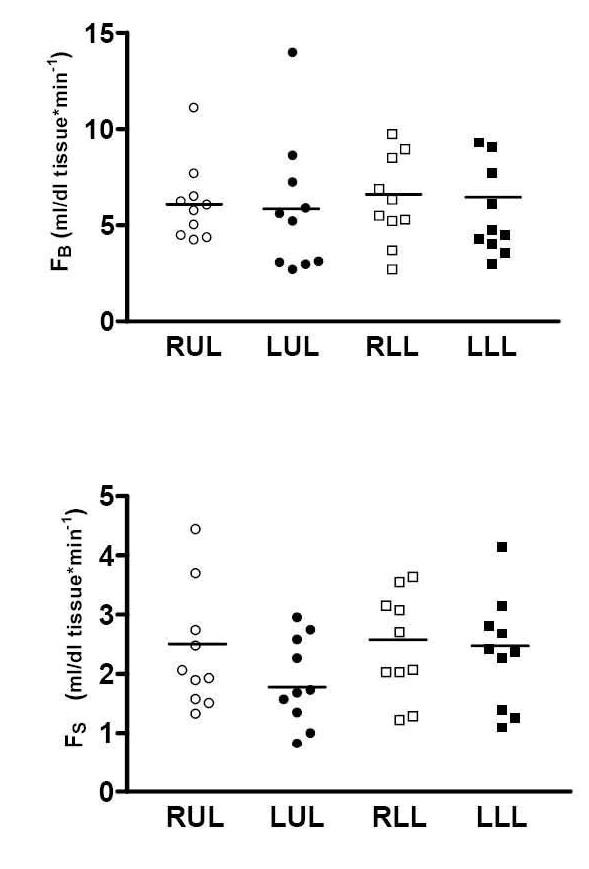
**Bone (upper graph) and soft tissue (lower graph) blood flow in each limb (RUL-right upper limb, LUL-left upper limb, RLL-right lower limb, LLL-left lower limb).** Blood flow units are expressed in ml/100 ml tissue·min-1 units, mean value for each column is marked with transverse line).

Blood flows in each limb and its compartments are presented in additional file 3 and in figure 2. F_B _was significantly higher compared to F_S _in all four limbs (6.16 ± 0.65 vrs 2.37 ± 0.30 in RUL, p < 0.001; 5.9 ± 1.1 vrs 1.87 ± 0.20 in LUL, p < 0.001;6.28 ± 0.72 vrs 2.48 ± 0.28 in RLL, p < 0.001; 5.63 ± 0.72 vrs 2.36 ± 0.29 in LLL, p < 0.001, units in ml blood per minute per 100 ml). No significant differences in either bone or soft tissue blood flows were measured between right and left limbs, both in the upper and the lower extremities.

## Discussion

Normal growth, remodeling and repair of bone require delivery of nutrients and oxygen through blood flow to bone tissue [21]. Interruption of normal bone and soft tissue blood flow has been shown to be responsible for the development of severe and common health problems including diabetic ulcers and osteoporosis [22]. Nevertheless, only limited literature is available on the physiology and pathophysiology of bone and soft tissue blood flow, as compared to other tissues (e.g. renal, brain) that have been thoroughly investigated. Presently, we describe a novel method that which enables noninvasive BBF quantification in humans.

Dual modality SPECT/CT imaging enables to quantify, with a high degree of precision, blood pool localized in a specific area of interest (in our study, the "soft" and "hard" tissues composing a limb). This method, combined with plethysmographic measurements, allows for quantification of blood flow in the tissues being evaluated. In this study, we showed that the BBF in the upper and lower limbs ranges between 5.5 and 6.5 ml per 100 ml of tissue per minute. These results are comparable with previously published data (e.g. Kubo et al., using ^15^O PET, showed that blood flow in femoral heads correlates with age and ranges between 1.7–6 ml/min per 100 g tissue) [23,5,4].

Data from animal studies using labeled microspheres reveals a variation in BBF, in the range of 5–20 ml/min per 100 g, within different regions of the same bone sample [10]. This method requires animal sacrifice for a direct measurement of fluorescence or radioactivity assessment, thus cannot be comparable to methods used in humans.

Our research has also shows that soft tissue blood flow (which is mainly a contribution of skeletal muscles) averaged between 1.87–2.48 ml/min per 100 ml tissue, which is comparable of PET measurements (range between 1.43–6.72 ml/min per 100 g muscle [5,4,24,2]. Noteworthy to mention that SPECT/CT-plethysmography revealed a trend towards a higher SBF in the dominant right upper limbs compared with the contralateral. Another interesting observation is that BBF was almost three times higher as compared to the adjacent SBF (per 100 ml tissue). Notice that while data in the literature is expressed as ml per minute per g tissue, ours is expressed as ml per minute per 100 ml tissue, since plethysmographic measurements are based on volume changes. This may be the reason for the small differences of our data from that described in the literature.

Venous-occlusive plethysmography is an easy and accurate method for the assessment of total limb blood flow. It cannot however, distinguish between the various tissue components in the limb. It cannot also differentiate between soft tissue components blood flow (i.e. skeletal muscle and skin). In this study, however, an anatomical CT interface such as CT was manually fused with data derived from SPECT studies in order to accurately localize blood flow measurements to the bone. Fusion methods of separately performed functional and structural imaging data are based, as a rule, on extrinsic or intrinsic landmarks. Accurate localization of these markers is, however, difficult and requires considerable operator skill. These drawbacks are more prominent in aligning the nuclear medicine data, which suffer from inherent low resolution. Inaccurate registration of separately acquired scintigraphic and CT data may be due to differences in patient positioning between studies, as well as to differences in organ location and volume at the time of imaging [18]. Sequential acquisition of scintigraphic SPECT and CT data during a single imaging session using SPECT/CT overcomes these limitations by accurate localization of blood pool, represented by uptake of labeled RBC, as demonstrated on SPECT, to specific areas in bone and soft tissues, as delineated by the CT.

### Study limitations and clinical perspectives

1. Plethysmography is a measurement technique that can only be applied to long bones and our method is, at present, only suitable for measuring limb blood flow. Future innovations involving a combination of SPECT/CT with different techniques for the assessment of regional blood flow (e.g., Dupplex) may allow for BBF measurement in flat/small bones.

2. Present method did not allow for separation of bone marrow from cortical bone flow. The use of improved devices with higher imaging resolutions may allow in the future studying the specific blood flow distribution within the bone.

3. When one comes to compare our results with those of the literature, he need to be aware that in the literature the bone blood flow results are presented in *ml blood per minute per 100 **grams **bone tissue*units. We, however, present the results in units of *100 ml blood per minute per 100 **ml **bone tissue*. In order to compare the values presented in the current paper with those in the literature, one need to correct the units that we used by dividing them in the specific gravity of the tissue. For example: we showed that the mean F_B _in the RUL is 6.16 ml blood per minute per 100 ml bone tissue. If the specific gravity of bone is (for example) 1.8 gr/ml, then the correction is 6.16/1.8 ml blood/min/100 gr bone.

We raised this points in order to precede one expected question regarding our results: the fact that we found bone blood flow much higher than soft tissue blood flow. If you correct our results using the specific gravity of each tissue, you will find them quite similar to those in the literature.

4. Our assumption that in resting-supine state is a steady state, where blood hydrodynamic characteristics between bone and muscle are comparable is essential, and the entire theory is based upon it. We could find neither support nor contradiction to this assumption in the literature, yet it seems only intuitive to us.

5. Our measurements cannot differentiate muscle from skin blood flow, thus SBF refers to both.

6. The resolution of the method, which supposedly determines a metric of blood flow in the bone, would be the smallest difference in blood flow this method can detect. The resolution is usually determined using a phantom simulating the procedure performed on the patient where all parameters are known. We do not believe we can determine this based on our method as no gold standard for osseous blood flow is currently available. The potential noise sources (factors that would influence the measured value that are not related solely to the blood flow in the bone) are:

a. Tecnical factors related to the veno-occlusive plethysmography (incorrect placement etc.)

b. Poor labeling of RBC.

c. Patient motion during acquisition of nuclear medicine study.

d. Misregistartion of CT and nuclear medicine portion of study due to motion.

e. Metallic devices in bone.

f. Operator error during processing of data.

## Conclusion

Bone blood flow is a physiologic characteristic that needs yet to be investigated in settings of clinical significances such as atherosclerosis, anti-hypertensive treatment, and osteoporosis, all conditions that are known to affect BBF. Here we offer it not as a replacement, but rather as additional method in the minute armamentarium of methods for BBF measurement.

## Competing interests

The authors declare that they have no competing interests.

## Authors' contributions

LD carried out the research designing, physiologic studies, data analysis and writing. ZK carried out the nuclear scan studies, participated in data processing and writing. OI participated in scan studies and data analysis. VM participated in data analysis. JS participated in scan studies and data analysis. GJ carried out the research designing, physiologic studies, data analysis and writing. All authors read and approved the final manuscript.

## Supplementary Material

Additional file 1 Clinical characteristics of subjects (presented as mean ± SEM). Click here for file

Additional file 2 Raw data extracted from SPECT/CT. Volumes (in ml) and scintigraphic readings (in counts) of total "cylinder" and bone. RUL-right upper limb, LUL-left upper limb, RLL-right lower limb, LLL-left lower limb. V_L _and R_L_-entire "cylinder" volume and counts, respectively. V_S _and R_S_, volume and counts of soft tissue, respectively. V_B _and R_B_, volume and counts of bone compartment, respectively. Data is expressed as mean ± SEM. Click here for file

Additional file 3 Blood flow measurements, resistance and ratios. Blood flow units are expressed in ml/100 ml tissue·min^-1 ^units. RUL-right upper limb, LUL-left upper limb, RLL-right lower limb, LLL-left lower limb. F_L_-total limb blood flow, F_B_-bone blood flow, F_S _blood flow in the soft tissue compartment. Data is expressed as mean ± SEM. Click here for file
