# Supplementary figures and images for: SPECT/CT-plethysmography – non-invasive quantitation of bone and soft tissue blood flow
Source: J Orthop Surg Res. 2008 Aug 18;3:36. doi: 10.1186/1749-799X-3-36 (PMC2527489; doi:10.1186/1749-799X-3-36)

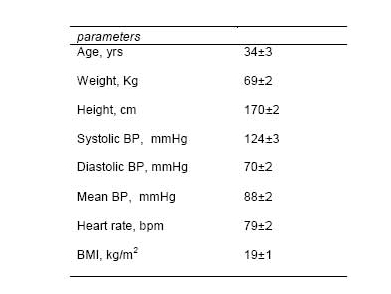

Supplement: Additional file 1 — Clinical characteristics of subjects (presented as mean ± SEM). [file 1749-799X-3-36-S1.jpeg]

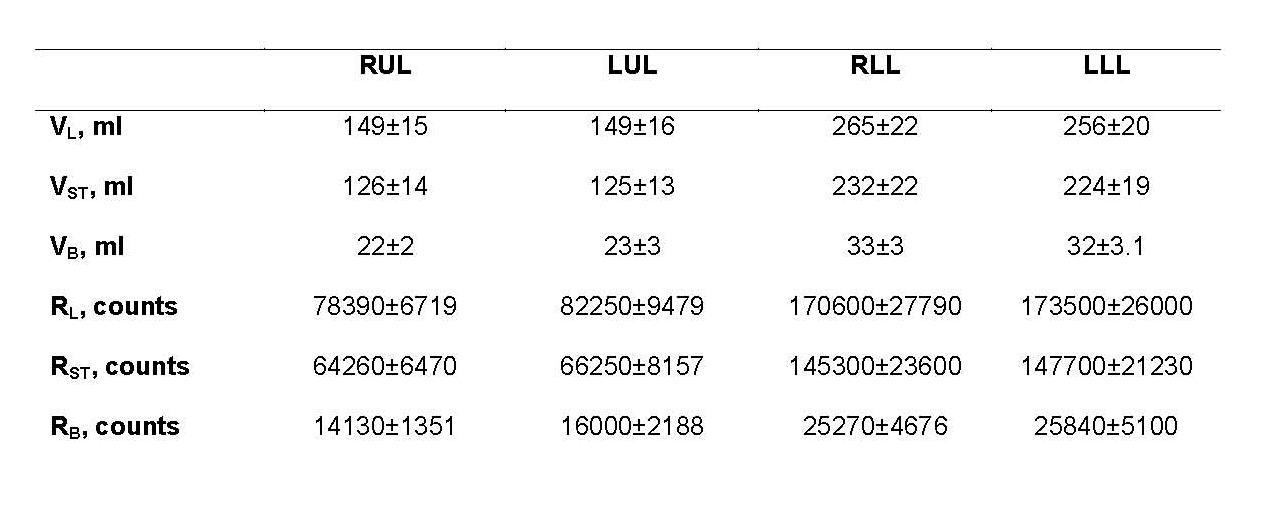

Supplement: Additional file 2 — Raw data extracted from SPECT/CT. Volumes (in ml) and scintigraphic readings (in counts) of total "cylinder" and bone. RUL-right upper limb, LUL-left upper limb, RLL-right lower limb, LLL-left lower limb. VL and RL-entire "cylinder" volume and counts, respectively. VS and RS, volume and counts of soft tissue, respectively. VB and RB, volume and counts of bone compartment, respectively. Data is expressed as mean ± SEM. [file 1749-799X-3-36-S2.jpeg]

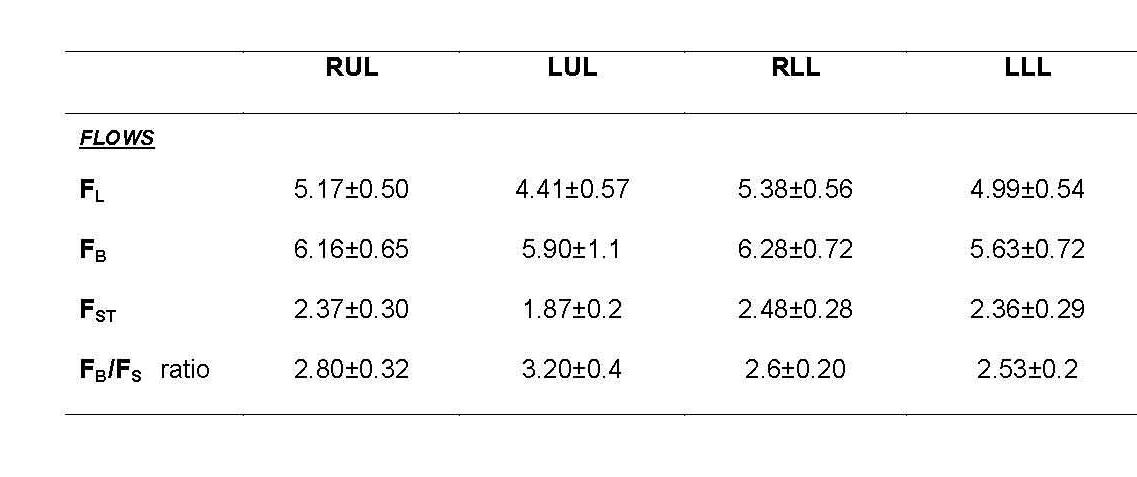

Supplement: Additional file 3 — Blood flow measurements, resistance and ratios. Blood flow units are expressed in ml/100 ml tissue·min-1 units. RUL-right upper limb, LUL-left upper limb, RLL-right lower limb, LLL-left lower limb. FL-total limb blood flow, FB-bone blood flow, FS blood flow in the soft tissue compartment. Data is expressed as mean ± SEM. [file 1749-799X-3-36-S3.jpeg]
